# Paraoxonase and Arylesterase Activities, Lipid Profile, and Oxidative Damage in Experimental Ischemic Colitis Model

**DOI:** 10.1155/2012/979506

**Published:** 2012-11-12

**Authors:** Ethem Unal, Cengiz Eris, Bülent Kaya, Hafize Uzun, Faruk Cavdar, Murat Yildar, Ali Riza Kiziler, Birsen Aydemir, Pembegul Gunes, Riza Kutanis, Izzet Titiz

**Affiliations:** ^1^General Surgery Clinic, Haydarpasa Numune Research and Training Hospital, Istanbul, Turkey; ^2^General Surgery Clinic, Fatih Sultan Mehmet Research and Training Hospital, Istanbul, Turkey; ^3^Department of Biochemistry, Cerrahpasa Medical Faculty, Istanbul University, Istanbul, Turkey; ^4^Deparment of General Surgery, Faculty of Medicine, Balikesir University, Balikesir, Turkey; ^5^Department of Biophysics, Faculty of Medicine, Namik Kemal University, Tekirdag, Turkey; ^6^Department of Biophysics, Faculty of Medicine, Sakarya University, Sakarya, Turkey; ^7^Department of Pathology, Haydarpasa Numune Research and Training Hospital, Istanbul, Turkey; ^8^General Surgery Clinic, Istanbul Bagcilar Research and Training Hospital, Istanbul, Turkey

## Abstract

*Objective*. In the present study, since PON1 is known as an HDL-associated antioxidant enzyme that inhibits the oxidative modification of LDL and oxidative stress plays a role in the pathogenesis of mesenteric ischemia, we investigated the changes in PON1 activity and lipid profile in an experimental ischemic colitis model. *Methods*. Forty male Wistar albino rats were divided into two groups: the control group (*N* = 15) and the experimental group (*N* = 25). All animals were anesthetized with ether and ketamine anesthesia to undergo a midline laparotomy. Ischemic colitis was induced by marginal vessel ligation in the splenic flexura (devascularization process). A sham laparotomy was performed in the control group. All animals were sacrificed on the seventh postoperative day. Oxidative stress marker (malonyldialdehyde, MDA), lipid profile, and paraoxonase (PON-1) and arylesterase activities were determined. Histopathological evaluation was done under light microscopy, after sectioning and staining with hematoxyline and eosin. Statistical analysis was conducted using Student's *t*-test and Mann-Whitney *U* test, and *P* < 0.05 was considered as statistically significant. *Results*. There was a significant decrease in both serum and tissue PON1 activity in ischemic colitis group (*P* < 0.01, for each). Similarly, arylesterase levels showed a parallel decrease in both tissue and serum of the experimental group (*P* < 0.01 and *P* < 0.001, retrospectively). MDA, an oxidative stress marker, was seen to increase in the experimental group (*P* < 0.01, tissue; *P* < 0.05, serum). In experimental group, there was a significant rise in serum total cholesterol and LDL levels (*P* < 0.001, for each). However, HDL level decreased significantly (*P* < 0.001). Triglycerides did not show any change between the groups (*P* > 0.05). *Conclusions*. PON1 and arylesterase play an important role in the pathophysiology of ischemic colitis.

## 1. Introduction

Intestinal ischaemia is caused by a reduction in intestinal blood flow. This entity is being increasingly recognised as a cause of abdominal symptoms but is often not diagnosed accurately. Chronic mesenteric ischaemia (intestinal angina) refers to chronic postprandial abdominal pain caused by intestinal hypoperfusion usually related to atherosclerosis [1]. Colonic ischaemia refers to colonic injury as a result of hypoperfusion and is also referred to as ischemic colitis.

Ischemic colitis is the most common form of intestinal ischemia and usually affects the left colon [2]. Common predisposing factors are atherosclerosis, shock, and congestive heart failure, but often elderly patients have no obvious predisposing or precipitating factors [3]. It is a disease complex that presents as a continuum of mucosal and submucosal hemorrhage, late stricture formation, and frank gangrene. The exact form depends upon the degree, site, and duration of the vascular occlusion, the presence of collateral vessels, and the intraluminal pressure in the colon.

Chronic mesenteric ischemia is a serious disease causing mortality, because of the poor understanding of its pathophysiology and its mild and nonspecific symptoms, which often delay its diagnosis [4]. Oxidative injury caused by free radicals is an important cause of tissue injury now recognized to occur in inflammation and ischemia. It is well known that free radical injury is counter balanced by antioxidants. The role of oxidative stress and lipid peroxidation in intestinal ischemia has been previously discussed in the literature [5, 6]. The data for the role of oxidative stress in the pathogenesis of ischemic, inflammatory, and radiation induced disease are strong, but interventional studies with antioxidants have shown only weak beneficial effects in the above diseases [5]. Therefore, the pathophysiology of the disease cannot be explained only by oxidative stress mechanisms.

Paraoxonase (PON1), the lipophilic antioxidant component of high-density lipoprotein (HDL) cholesterol, has been shown to reduce the susceptibility of LDL to lipid peroxidation [7]. PON1, with its antioxidant activity, protects lipoproteins against oxidation, probably by hydrolyzing specific lipid peroxides [7, 8].

Although the role of lipid peroxidation in mesenteric ischemia has been reported [5, 6], there is no study on the changes of PON1 with particular emphasis on the lipid profile and oxidative stress in ischemic colitis.

In the present study, since PON1 is known as an antioxidant enzyme that inhibits the oxidative modification of LDL and oxidative stress plays a role in the pathogenesis of mesenteric ischemia, we investigated the changes in PON1 activity and lipid profile in an experimental ischemic colitis model.

## 2. Material and Method

The study was conducted with the approval of our institutions' ethics committee, and all experimental procedures were done according to the standards of ACUC [9]. Forty male Wistar albino rats weighing 250–320 g were divided into two groups: the control group (*N* = 15) and the experimental group (*N* = 25). The animals were fed on standard laboratory diet and water ad libitum before and after surgery. 

**Figure 1:**
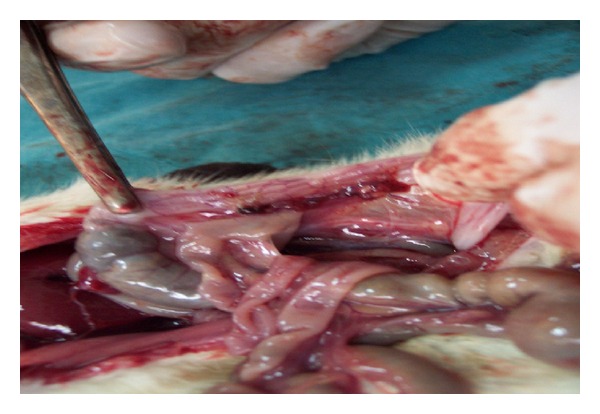
Devascularization procedure.

All animals were anesthetized with ether and ketamine (intraperitoneal 50 mg/kg injection) anesthesia to undergo a midline laparotomy. Ischemic colitis was induced by marginal vessel ligation in the splenic flexura (devascularization process) (Figure 1). A sham laparotomy was performed in the control group.

All animals were sacrificed on the seventh postoperative day. Under ether anesthesia, 4 cm^3^ (3–7 cm^3^) of blood was taken by cardiac puncture after exploration of the thorax. Then, a laparotomy was done and the devascularized segment of the colon was excised. The corresponding colonic segment was removed in the control group. The excised segments were fixed in 10% formol solution for histopathologic confirmation.

### 2.1. Biochemical Analyses

Blood samples collected in vacutainer tubes were immediately transported to the laboratory in a cooler with ice. Upon arrival, serum was separated by centrifugation (+4°C, 3000 rpm, 10 minute) and divided into 0.5–1 mL. aliquots, placed in cryovials, and stored at −70°C until being analyzed. Each serum sample was divided into 3 aliquots; lipids were studied immediately in 1st aliquot; 2nd aliquot was saved until analysis of serum PON1 and arylesterase within 2 weeks, and 3rd aliquot was used for estimation of plasma malondialdehyde (MDA).

#### 2.1.1. Assay of TBARS (Thiobarbituric Acid-Reactive Substances)

Lipoperoxidation was ascertained by the formation of MDA, which was estimated by the modified thiobarbituric acid method, described by Buege and Aust [10]. TBARS concentration was calculated using 1.56 × 10^−5^ M^−1^ cm^−1^ as mol/L extinction coefficient.

#### 2.1.2. Assay of PON1 Activity

PON1 activity was assayed using synthetic paraoxon (diethyl-p-nitrophenyl phosphate) as substrate. PON1 activity was determined by measuring the initial rate of substrate hydrolysis to p-nitrophenol, which absorbance was monitored at 412 nm in the assay mixture containing 2.0 mM paraoxon, 2 mM CaCl_2_, and 20 *μ*L of plasma in 100 mM tris-HCI buffer (pH 8.0). The blank sample containing incubation mixture without plasma was run simultaneously to correct for spontaneous substrate breakdown. The enzyme activity was calculated from E412 of p-nitrophenol (18.290 per M/cm) and was expressed as U/mL.

#### 2.1.3. Arylesterase Activity

Arylesterase enzyme activity was measured at 270 nm spectrophotometry, by determining phenylacetate hydrolysis. Tris-HCL tamponade (3 mL) added 1 mM CaCl_2_ and 1 milieu. Arylesterase concentration was calculated using 1310 M^−1^ cm^−1^ (pH: 8) molar extinction coefficient. The results were expressed as U/mL.

#### 2.1.4. Assay of Lipid Profile

Other serum parameters (total cholesterol, triglycerides, and HDL, LDL cholesterol) were determined by routine laboratory methods using the Hitachi 704 autoanalyzer (Boehringer Mannheim, Tokyo, Japan).

#### 2.1.5. Preparation of Tissue Samples

About 190–200 mg of each intestine tissue sample was weighed and diluted 20% w/v in 20 mM ice-cold Tris-HCl, pH 7.4 and homogenized with a Bosch Scintilla SA (Switzerland). The homogenate was centrifuged at 5000 ×g for 10 min, and biochemical parameters were performed in the supernatant fraction.

#### 2.1.6. Determination of Malondialdehyde (MDA)

Lipoperoxidation was ascertained by the formation of thiobarbituric acid reactive substances (TBARS) (MDA), which was estimated by the modified thiobarbituric acid method, described by Buege and Aust [1]. MDA concentration was calculated using 1.56 × 10^−5^ M^−1^ cm^−1^ as mol/L extinction coefficient. The results were expressed as *μ*mol/L.

#### 2.1.7. Determination of PON1 Arylesterase Activity

Arylesterase activity was also measured spectrophotometrically using phenylacetate (Sigma Co., London, UK) as the substrate. The assay mixture contained 100 *μ*L of 10 mmol/L substrate solution, 5 *μ*L serum, and 1 mmol/L CaCl_2_ (Sigma, USA) in 50 mmol/L Tris buffer (Fluka Chemie, Switzerland), pH = 8. Production of phenol was determined spectrophotometrically after 2 min at 270 nm. The assay mixture was prepared daily before use. PON1 arylesterase activity was monitored in triplicate and the results are presented as *μ*mol/min per mL [2]. Mean intraassay and interassay coefficients of variation were up to 4.3% and 5.6%, respectively.

#### 2.1.8. Determination of PON1 Paraoxonase Activity

PON1 activity was assayed using synthetic paraoxon (diethyl-p-nitrophenyl phosphate) as substrate. PON1 activity was determined by measuring the initial rate of substrate hydrolysis to p-nitrophenol, which absorbance was monitored at 412 nm in the assay mixture containing 2.0 mM paraoxon, 2.0 mM CaCl_2_ and 20 *μ*L of plasma in 100 mM Tris-HCI buffer (pH = 8.0). The blank sample containing incubation mixture without plasma was run simultaneously to correct for spontaneous substrate breakdown. The enzyme activity was calculated from E412 of p-nitrophenol (18.290 per M/cm) and was expressed as U/mL; 1 U of enzyme hydrolyses 1 nmol of paraoxon/min [3]. Mean intraassay and interassay coefficients of variation for this analysis were 4.2 % and 6.1%, respectively.

### 2.2. Histopathological Evaluation

Histopathological evaluation was done under light microscopy, after sectioning and staining with hematoxyline and eosin.

### 2.3. Statistical Analyses

All values are expressed as the mean ± SD. Statistical analysis was conducted using Student's *t*-test and Mann-Whitney *U* test by SPSS statistical software package (SPSS Inc., Chicago, USA). *P* < 0.05 was considered as statistically significant. 

## 3. Results

**Figure 2:**
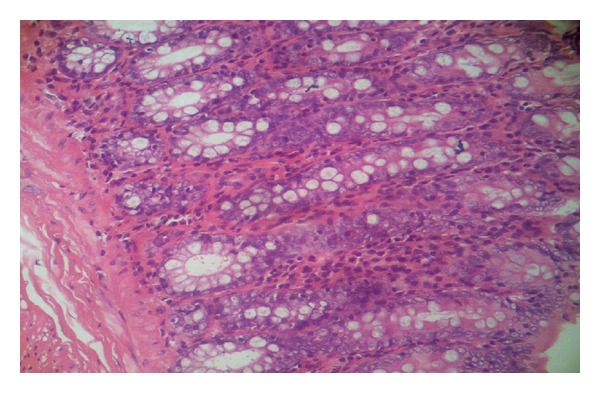
Normal colonic mucosa (H&E, ×200).

**Figure 3:**
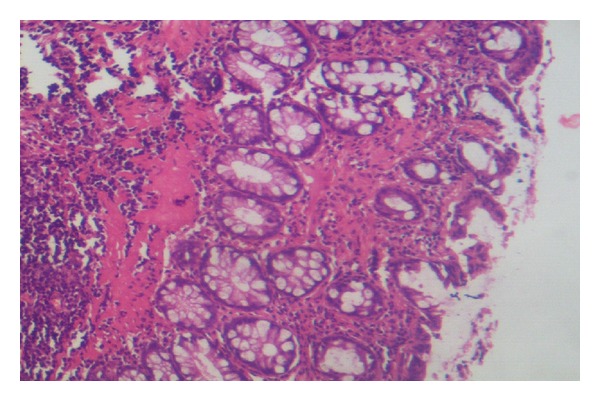
Irregular crypt, fibrosis, lymphoplasmacytic infiltration (H&E, ×200).

Histopathological studies confirmed our ischemic colitis model induced by devascularization procedure. In comparison to control group (Figure 2), experimental group showed cryptal irregularities, mucin loss, and focal fibrosis in the colonic mucosa. There was also eosinophilic and lymphoplasmacytic infiltrations in the lamina propria (Figure 3).

Values of the analyzed parameters and the statistical significances in the groups are shown in Tables 1 and 2. 

There was a significant decrease in both serum and tissue PON1 activity in ischemic colitis group (*P* < 0.01, for each). Similarly, arylesterase levels showed a parallel decrease in both tissue and serum of the experimental group (*P* < 0.01 and *P* < 0.001, retrospectively). The significant drop in serum arylesterase activity was outstanding (*P* < 0.001). MDA, an oxidative stress marker, was seen to increase in the experimental group (*P* < 0.01, tissue; *P* < 0.05, serum).

In experimental group, there was a significant rise in serum total cholesterol and LDL levels (*P* < 0.001, for each). However, HDL level decreased significantly (*P* < 0.001). Triglycerides did not show any change between the groups (*P* > 0.05). 

The parallel drop in the serum levels of HDL and arylesterase was significant (*P* < 0.001, for both).

## 4. Discussion

Ischemic colitis is one of the most often seen disorders of the large intestine in the elderly. The typical clinical presentation is acute sudden abdominal pain and distention with bloody diarrhea. Common early radiographic signs are bowel-wall thickening with thumbprinting, and later, ulceration and strictures may be found [1, 11]. The predominant predisposing causes are atherosclerosis, shock, and congestive heart failure [3]. The absence of colonic infarction does not ensure a favorable outcome. Patients who are felt to be candidates for nonoperative therapy have significant mortality rates. Mortality rates remain high, despite treatment [4].

In the present study, we investigated the possible changes in PON1 activity and lipid profile with particular emphasis on the oxidative stress in an experimental ischemic colitis model. 

Oxidative injury caused by free radicals is an important cause of tissue injury now recognized to occur in inflammation and ischemia. The role of oxidative stress and lipid peroxidation in intestinal ischemia has been previously discussed in the literature [5, 6]. The data for the role of oxidative stress in the pathogenesis of ischemic and inflammatory diseases of the colon are strong, but interventional studies with antioxidants have shown only weak beneficial effects [5]. Therefore, the pathophysiology of the disease cannot be explained only by oxidative stress mechanisms. Our data have confirmed the role of oxidative stress in the pathogenesis of ischemic colitis. According to our findings, MDA as an oxidative stress marker was seen to increase in both tissue and serum of the experimental group.

PON1 is an HDL-associated antioxidant enzyme that inhibits LDL cholesterol oxidation in human serum [7, 8]. PON1 confers protection against free radicals by limiting the oxidation of phospholipids and is known to lose its activity in the oxidative environment [12, 13]. As atherosclerosis and oxidative stress are known to be involved in the pathogenesis of ischemic colitis, in the present study, we investigated the possible changes in PON-1 activity in an experimental model of ischemic colitis, for the first time in the world literature.

Several recent studies have suggested that PON1 concentration decreases in some inflammatory and ischemic diseases, such as diabetes and acute pancreatitis, which are associated with an increase in oxidative stress [14, 15]. In these studies, Mackness et al. have shown that low PON1 activity may contribute to the increased atherosclerosis found in type 1 diabetes by reducing the ability of HDL to retard LDL oxidation [14]. Similarly, Unal et al. have found that PON1 activity significantly decreased in acute pancreatitis with a positive correlation to the serum HDL level, while there was a significant increase in the oxidative stress agent, MDA [15]. 

There are other studies showing the relationship between paraoxonase activity and coronary artery disease (CAD). Granér et al. have indicated that PON1 activity and concentration are lower in subjects with significant CAD and that there is a significant relationship between PON1 activity and concentration and CAD assessed by coronary angiograpy [16]. Furthermore, Manresa et al. have established the nonclassical risk factors of coronary heart disease as lipid status, inflammation, PON1, and oxidative stress [17].

In our study, there was a significant decrease in both serum and tissue PON1 activity in ischemic colitis group. Similarly, arylesterase level showed a parallel decrease in both tissue and serum of the experimental group. Moreover, the significant drop in serum arylesterase activity was outstanding (*P* < 0.001). The parallel drop in the serum levels of HDL and arylesterase was also significant (*P* < 0.001, for both). These findings absolutely confirmed the association between HDL cholesterol and PON1-arylesterase enzymes. At this point, it can be hypothesized that the pathogenesis of ischemic colitis is associated with increased oxidative stress and impaired HDL-associated antioxidant defense, evidenced by decreased paraoxonase and arylesterase activities. Furthermore, the significant rise in serum total cholesterol and LDL levels supports this hypothesis, regarding the role of atherosclerosis in the pathophysiology of ischemic colitis. 

In conclusion this study has some weak points. It is an animal study. Translation of the results from rats to humans can be difficult. Anyway our findings indicate that lower serum paraoxonase and arylesterase activities may be associated with lipid metabolic disorders and oxidative damage in ischemic colitis. A decreased serum paraoxonase activity may show the ineffective antioxidative capacity of the body, resulting in ischemic symptoms.

## Figures and Tables

**Table 1:** PON1 and arylesterase activities and lipid peroxidation marker level in control and experimental (ischemic colitis) groups.

Groups	Tissue/PON1 activity U/wet tissue gr	Tissue/MDA nmol/wet tissue gr	Tissue/arylesterase activity U/wet tissue gr	Serum/ PON1 activity U/mL	Serum/ MDA nmol/L	Serum/ arylesterase activity U/mL
Control (*N* = 15)	87.84 ± 21.49	18.26 ± 8.70	14.34 ± 8.58	252.61 ± 100.68	3.86 ± 1.52	134.52 ± 40.17
Experimental (*N* = 25)	68.01 ± 19.01	30.06 ± 13.29	9.11 ± 4.56	154.74 ± 79.34	5.48 ± 2.37	70.62 ± 21.18
*P*	<0.01	<0.01	<0.05	<0.01	<0.05	<0.001

**Table 2:** Lipid profile.

	Control group	Experimental group
	(*N* = 15)	(*N* = 25)
Total cholesterol (mg/dL)	114.2 ± 31.8	141 ± 39.03*
Triglycerides (mg/dL)	69.1 ± 17.3	71.81 ± 20
HDL (mg/dL)	39 ± 9.4	21 ± 3.9*
LDL (mg/dL)	53.5 ± 19.9	91 ± 48.21*

*P < 0.001.
